# On the Importance of Correct Snake Identification. Comment on Chippaux et al. Snakebites in Cameroon by Species Whose Effects Are Poorly Described. *Trop. Med. Infect. Dis.* 2024, *9*, 300

**DOI:** 10.3390/tropicalmed11010031

**Published:** 2026-01-22

**Authors:** Wolfgang Wüster, David A. Warrell, David J. Williams

**Affiliations:** 1 School of Environmental and Natural Sciences, Bangor University, Bangor LL57 2UW, UK; 2 Nuffield Department of Clinical Medicine, University of Oxford, John Radcliffe Hospital, Headley Way, Headington, Oxford OX3 9DU, UK; david.warrell@ndm.ox.ac.uk; 3 Regulation and Prequalification Department, World Health Organization, Avenue Appia 20, CH-1211 Geneva, Switzerland; williamsd@who.int

One of the major obstacles to improving the management of snakebite envenoming is the lack of accurate identification of species responsible for clinical cases, which prevent the improvement of definitions of species-specific syndromes. Understanding which snakes are biting people in a given region of the world, and what the clinical consequences of these cases are, is a cornerstone to the formulation of strategies to reduce the burden of snakebite. Yet, in many parts of the world, this knowledge remains elusive. Nowhere is this problem more acute than in the forested zones of Central and Western Africa, where detailed epidemiological studies remain conspicuous by their absence, the key species involved in severe envenoming remain largely unknown, and their syndromes poorly understood and ill-defined. It is thus with considerable excitement that we read the recent paper by Chippaux et al. [1], promising a description of the syndromes of envenoming by several rarely documented snake species in Cameroon.

However, we were troubled to find that upon comparing clinical notes and photographs, there were multiple misidentifications of specimens to which cases were attributed. Coupled with clinical incongruities, these inaccuracies cast considerable doubt on the value of the paper.

The following are clear misidentifications of the snakes shown in the photographs:

- Figure 7 [1]: the snake shown is unquestionably a *Naja nigricollis*, not a *N. haje*. An enhanced version of the Figure is shown in our Figure 1A. Its identity is evident from the clearly visible second (lower) preocular, the contact between the 3rd supralabial and the eye (Figure 1A—the scale contacting the eye is clearly contiguous with the 3rd supralabial, although there may be a small surface irregularity conveying an impression of partial separation), and the small penultimate supralabial, all characters that distinguish African spitting cobras (subgenus *Afronaja*) from the *Naja haje* group (subgenus *Uraeus*) [2,3,4]. The temporal and supralabial scalation is more challenging to discern. Superficial examination gives the impression of a single anterior temporal, bordered below by a high supralabial that is also in broad contact with the postoculars. However, closer inspection suggests that the lower supralabial edges are curled inwards towards the mouth, and that what looks like a high supralabial is in fact a lower temporal, bordered below by the typical low 4th and 5th supralabials of *Afronaja* spp. that are, in this photo, largely hidden by the overhanging temporal region (Figure 1A). A corner of the low 4th supralabial is just about visible between the posterior edge of the third supralabial, the anterior edge of the lower anterior temporal and the edge of the mouth. Additionally, the illustrated specimen has approximately square internasals in broad contact with each other and a low rostral scale, whereas in *N. haje,* the rostral is high and separates the anterior inner edges of the internasals from each other. This identification is also supported by the clinical syndrome of envenoming that consisted of massive swelling of the entire bitten arm with large blisters on the dorsum of the hand, in the absence of any neurotoxic signs. The patient died 30 h after the bite, possibly from hypovolaemic shock as there is no mention of fluid replacement being given. This clinical picture is consistent with *N. nigricollis* but not with *N. haje* envenoming, which entails significant neurotoxicity that can sometimes be accompanied by local swelling and minor blistering, but not cytotoxicity without neurotoxic signs [5,6].

- Figure 8 [1]: the snake shown is almost certainly a *Naja nigricollis*. In *N. katiensis*, the underside of the head and the first 8–15 ventral scales are uniformly light, followed by 1–2 narrow dark bands, followed by an immaculate light belly, whereas the overwhelmingly dark throat and chin and the numerous black markings on otherwise light ventral scales in the snake in Figure 8 are indicative of *N. nigricollis*. In many western and central African *N. nigricollis*, the head and throat are entirely black, but in some, including in northern Cameroon (Figure 1B), the underside of the head and the first few ventrals can be light. The width of the first dark ventral band is an additional distinguishing character: in 13 published [3,4,8,9,10,11,12] or Internet photographs of *N. katiensis,* the width of the first band ranged from 4–8 ventrals compared to the 11 ventrals in Chippaux et al.’s Figure 8. In addition, *N. katiensis* has more numerous dorsal scale rows (typically 25) compared to *N. nigricollis* (typically 21) [4,13,14,15], resulting in a smoother, shinier appearance than *N. nigricollis*. This is clearly not the case in the snake depicted in Figure 8. The dark edges of the dorsal scales mentioned by Roman [14] for *N. katiensis* are not visible in any widely available published photo [3,4,8,10,11,12,16]. Finally, the frontal scale of the specimen in Figure 8 is approximately as broad as long, corresponding to the typical condition in *N. nigricollis*, whereas in *N. katiensis*, it is typically much longer than broad [3,14]. Unfortunately, details of the temporal and infralabial scalation of the specimen are not discernible due to the poor resolution of the photo in the published article. The largely light belly of the specimen in Figure 8 is somewhat unusual for *N. nigricollis*. However, given the dark rectangular markings along the belly and the numerous features listed above, we argue confidently that this specimen is *N. nigricollis*.
- Figure 11 [1]: there are multiple misidentifications in this Figure.
  ○ Figure 11A [1] depicts *Dendroaspis jamesoni*, not *D. viridis*, a species not found in Cameroon, and can be distinguished by its conspicuously enlarged upper dorsal scales; as *D. viridis* is not otherwise mentioned in [1], we assume that this is an unfortunate clerical error.
  ○ Figure 11B [1] depicts a *Causus* sp., certainly not *Bitis arietans*; the specimen displays none of the typical pattern elements of *B. arietans*, such as the series of light chevrons along the back. The narrow, angular dark marks suggest *C. resimus*, which is not normally green in this part of its distribution [4].
  ○ Figure 11C [1] depicts *Bitis arietans*, not *Echis romani*, as it lacks the characteristic vertebral blotches and lateral ocelli of the latter, but displays typical pattern elements of *B. arietans*.

In summary, 5 out of 12 snakes (42%) shown in the photos in this paper are unambiguously misidentified, a ratio worsened by the fact that several of the remaining photos do not show the level of detail required for a species-level identification, either in the PDF file of the article or in the HTML version on the journal website. If this high rate of misidentification, which compares unfavourably with the correct identification rates achieved by crowdsourcing snake identifications from photos [17], were to be extrapolated to the other snake identifications in the article, it would cast grave doubts on the reliability of the entire paper. Importantly, the lack of locality information for individual cases impedes the verification of records where the snake cannot be confidently identified, and represents a wasted opportunity to provide more geospatially and herpetologically detailed epidemiological information.

The issues highlighted here point to the wider problems of misidentifications in the clinical snakebite literature [18]. Photos of snakes responsible for bites, taken either at the bedside or by the victim or their companions at the time of the accident, represent valuable vouchers allowing for the verification of identifications in studies such as this, and have allowed for the reidentification of snakes in other studies, in some cases leading to retractions (e.g., [10], where the snake responsible was a *Trimeresurus purpureomaculatus* rather than *Rhabdophis subminiatus*). However, even when misidentifications can later be corrected, the damage may have been done. For instance, because of the paper discussed here, the literature now contains a false record of a fatal cytotoxic envenomation by *Naja haje*, potentially misleading future work.

As always, the publication of inaccurate scientific information suggests a failure to exercise due diligence by both the authors and the editorial review process of the journal. To maintain the integrity of the scientific record, we recommend that editors of scientific journals should send all manuscripts involving snake identifications to qualified herpetologists for review, even when one or several authors are themselves widely acknowledged snake and snakebite experts, as is the case here. Also, since multiple misidentifications in the figures shown in manuscripts erode confidence in the identification of bite cases where the snake is not depicted, we suggest that studies of this nature should always provide, in a supplementary online appendix, all photos used for the identification of snakes responsible for bites, at full original resolution and with locality details, all unfortunately absent in the paper discussed here. Ideally, the photos should show the upper and lower sides of the head and neck, both sides of the head (in case of anomalies), and the entirety of the ventral and dorsal surface of the snake. Deposition of photos in natural history museums and online databases such as iNaturalist or HerpMapper will add further to their value. Where possible under local circumstances, the collection of tissue samples for DNA analysis and the deposition of specimens in natural history collections will allow for further verification of the material and thus future-proof the output of epidemiological studies. These suggestions will increase both the verifiability of the data and add valuable information on the distribution of medically important species and on the epidemiology of snakebite, and thereby prevent erroneous information from entering and being disseminated through the scientific literature.

## Figures and Tables

**Figure 1:**
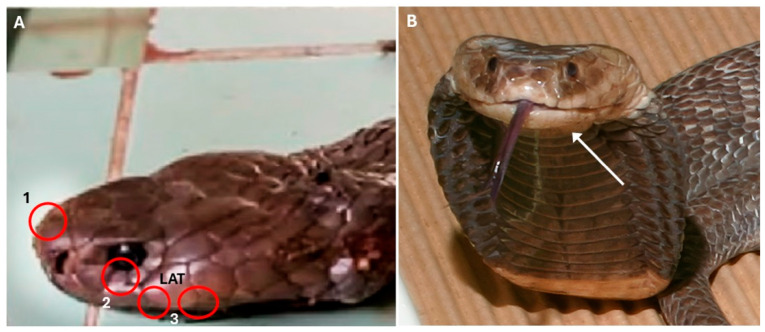
(**A**). Enhanced version of Figure 7 from [1], showing typical features of *Naja nigricollis*. 1: squarish internasals without intrusion of upper rostral scale edge; 2: 3rd supralabial enters eye; 3: low 4th and 5th supralabial scales can be seen protruding from below the lower anterior temporal (LAT), showing that it is not a supralabial. Although the photo was enhanced, these features can also be seen in the original paper. (**B**) Specimen of *Naja nigricollis* from Kaélé, northern Cameroon, demonstrating light underside of head and light first few ventrals (white arrow), as also seen in Figure 8 of [1]. This individual (Latoxan live collection, N. ni. ssp. 9735002) was sequenced and included in [7].
